# Evaluation of the role of Care Sport Connectors in connecting primary care, sport, and physical activity, and residents’ participation in the Netherlands: study protocol for a longitudinal multiple case study design

**DOI:** 10.1186/s12889-015-1841-z

**Published:** 2015-11-23

**Authors:** E. Smit, K.E.F. Leenaars, M.A.E. Wagemakers, G.R.M. Molleman, M.A. Koelen, J. van der Velden

**Affiliations:** Academic Collaborative Centre AMPHI, Primary Health Care, Radboud university medical center, P.O. Box 9101, , 6500 HB Nijmegen, The Netherlands; Department of Social Sciences, Health and Society Group, Wageningen University & Research Centre, P.O. Box 8130, Wageningen, The Netherlands

**Keywords:** Care Sport Connector, Health broker, Primary care, Sport, Physical activity, Intersectoral collaboration

## Abstract

**Background:**

The number of people with one or more chronic diseases is increasing, but this trend could be reduced by promoting physical activity. Therefore, in 2012, the Dutch Ministry of Health, Welfare, and Sport introduced Care Sport Connectors (CSCs), to whom a broker role has been ascribed. The defined outcome of CSCs role is an increased number of residents participating in local sports facilities and being physically active in their own neighbourhood. To realize this, primary care and sports professionals need to collaborate, and local sports facilities and neighbourhoods need to offer accessible physical activities for people in the locality, including people with one or more chronic diseases or at increased risk of chronic disease(s). Adequate scientific research is needed to assess CSCs’ impact on: 1) connecting primary care, sport, and physical activity and 2) increasing the number of residents who engage in physical activity to promote their health.

**Methods and design:**

To study the role and the impact of CSCs, a longitudinal multiple case study will be conducted, in nine municipalities spread over the Netherlands, from 2014 until 2017. A mixed methodology will be used to perform action research and process evaluation. Study I focuses on the expected alliances of CSCs and the preconditions that facilitate or hinder CSCs in the formation of these alliances. The study population will consist of intermediary target groups. A literature review, interviews, focus groups, and document analysis will be undertaken. Study II will concentrate on lifestyle program participants to identify health and physical activity behavior changes. For this purpose, interviews, literature studies, a Delphi study, fitness tests, and questionnaires will be used.

**Discussion:**

Linking and integrating results gained by multiple methods, at different levels, will provide a validated assessment of CSCs’ impact on connecting the primary care and sports sectors. This will reveal changes in residents’ physical activity behavior, and also the circumstances under which this will happen. The assessment in combination with general lessons learned from the different case studies will make it possible to determine whether CSCs are able to fulfill the policy aspiration and whether it would be beneficial to extend this function.

**Trial registration:**

Nederlands Trialregister NTR4986. Registered 14 December 2014.

## Background

Physical activity is recognized as one of the main determinants of health because of its numerous benefits for the musculoskeletal, cardiovascular, metabolic, endocrine, psychological and immune systems [1, 2]. In the Netherlands, 32 % of the total population is diagnosed with one or more chronic diseases, and this figure is expected to increase to 40 % by 2030 [3]. Only slightly more than 50 % of this group is meeting the Dutch Healthy Physical Activity Guideline [4], i.e., 30 min of moderate physical activity at least five days a week. A significant reduction in the percentage of the population contracting one or more chronic diseases is possible by tackling and preventing physical inactivity, which is the fourth leading independent risk factor for death caused by non-communicable chronic disease [5]. Increasing physical activity is a challenge because of different and interrelated determinants that contribute to lifestyle behaviors at multiple levels such as the individual, the social, the environmental, and the policy level [6]. Therefore, an ecological approach is most appropriate to address physical activity behavior [7].

In order to develop activities to promote health, it is necessary that different actors, both within and outside the health sector, collaborate with one another [8–10]. This intersectoral collaboration often takes place in the form of alliances or networks. Through intersectoral collaboration, talents, resources, relations, and approaches to influencing determinants of health can be linked and shared to work very much more effectively, efficiently, and sustainably than one sector would achieve alone [8–12]. Despite the fact that intersectoral collaboration is more effective and efficient to reach health goals, it is quite difficult to build effective and sustainable partnerships [12–14]. The fact that each actor and sector has different backgrounds, interests, perspectives, cultures, and knowledge domains makes collaboration challenging [12, 14] and not always successful [15, 16]. A Dutch study demonstrated that building alliances between the primary care sector and the sports sector to initiate and implement the BeweegKuur, a combined lifestyle intervention, was hampered because each sector had different cultures and different target groups [17].

Another difficulty, revealed by this study on the BeweegKuur and in other studies, is that residents’ participation in the interventions is hard to realize [17–20]. Moreover, it is challenging to motivate people, especially people with health problems, to participate in physical activities [3, 21], although, based on a literature review [22], there is evidence that primary care-based physical activity interventions are effective in reaching physically inactive adults. Another barrier, faced by lifestyle interventions aiming to transfer patients from primary care to local physical activity facilities, is that transferal levels often lag behind desired levels [17–19]. Transferal of patients is limited because, amongst other things, patients prefer to stick in the known and secure environment of the primary care sport facilities instead of participating in unknown or untried local facilities [19, 23]. The above indicates that both participation and transferal rates are much lower than expected; this might mean that patients are not sufficiently prepared for the responsibilities of self-management [23]. Self-management refers to the ability of a person to cope with a disease or the possible risk factors, and to the personal skills to maintain or improve health and wellness [24]. Apparently, patients need to be better equipped to manage their own physical activity behavior during and after an intervention. For patients to become more confident and motivated to be physically active, they probably need the support of a healthcare professional [25, 26]. A previous study indicates that better results are achieved when professionals personally direct patients to local sports facilities [19]. However, the major time investment by primary care professionals that this requires is impossible for the majority of them [27].

To address the described challenges, to improve collaboration between sectors, and to increase patients’ participation and self-management, a broker role seems to be promising. Previous studies have revealed that a broker role improves collaboration between multiple sectors [28, 29]. A broker with the task of connecting the primary care and sports sectors, is in the position to support professionals in developing and implementing activities that stimulate patients to participate and transfer to local sports facilities, because they have contacts with both sectors [27]. Therefore, in 2012, the Dutch Ministry of Health, Welfare, and Sport introduced Neighborhood Sports Coaches (*Buurtsportcoaches*), to whom a broker role has been ascribed. This function is 40 % funded by the state, with the remaining 60 % funded by the municipality or other local organizations. Several Neighborhood Sport Coaches focus specifically on the connection between primary care, sport, and physical activity, the so-called Care Sport Connectors (CSCs). The defined outcome of the CSC role is an increased number of residents participating in local sports facilities and being physically active in their own neighbourhood. To realize this, local sports facilities and neighbourhoods need to offer accessible physical activities for people in the neighbourhood, including people with one or more chronic diseases or at increased risk of chronic disease(s) [30]. The general idea is that CSCs facilitate the connection between the primary care, sport, and physical activity sectors; professionals in these sectors collaborate; these professionals implement lifestyle interventions; the lifestyle interventions reach certain target groups; these target groups will become self-manageable regarding their physical activity; target groups will become more physically active in their neighborhood; and health outcomes will improve.

The introduction of the CSC concept is new and unique. However, there is no clear job description with required competencies or a clear idea of how CSCs can be embedded in their context. As far as can be ascertained, to date there have been no in-depth studies about a health broker role that reveal the specific competencies that go with the role, the impact of the role on connecting different sectors, and on residents’ health. It is accepted that CSCs will operate in different ways because of their different backgrounds and different contexts. Adequate scientific research is needed to assess CSCs’ impact on: 1) connecting primary care, sport, and physical activity and 2) promoting the health of primary care patients. This research project consists of two studies to get insight into the impact of the CSC function and into opportunities and lessons to advance health promotion theory and practice in the Netherlands.

Study I focuses on the intermediary target groups: CSCs and professionals active in primary care, sport, and physical activity who implement lifestyle programs. CSCs are expected to form health alliances by connecting professionals from different sectors and to achieve and sustain collaboration in these alliances. Consequently, the following research questions will be examined:

1. What are the processes that contribute to the connection between primary care, sport and physical activity, and what is the role of the CSC in forming these alliances?
2. What are the conditions at national and local level that facilitate or hinder CSCs in connecting primary care, sport, and physical activity?
3. Which impacts are mediated by CSCs, and what are the perceived societal benefits for the municipality, neighborhood, and local residents?

Study II concentrates on health and physical activity behavior changes of primary care patients who participate in lifestyle programs. Center of attention is the target group: adults from the neighborhood who participate in lifestyle programs organized by professionals from the alliances of Study I. The following research questions will be addressed:

1. Which lifestyle programs are implemented, and which target groups are reached?
2. What strategies are effective in increasing participation, self-management and transferal of primary care patients, and which preconditions are essential to accomplish these?
3. What is the effect in terms of physical activity behavior and maintenance, self-management, quality of life, experienced health, and health gains?

### Theoretical framework

To frame this study and the research questions, it is necessary to get insight into the context in which CSCs work and how behavior change may take place within this context. Therefore, the theoretical framework is based on the Expanded Chronic Care Model (ECCM) [24] and the Theory of Triadic Influence (TTI) [31]. This framework will be used to position the function of a CSC and individual behavior changes from a holistic perspective.

#### The Expanded Chronic Care Model

**Fig. 1:**
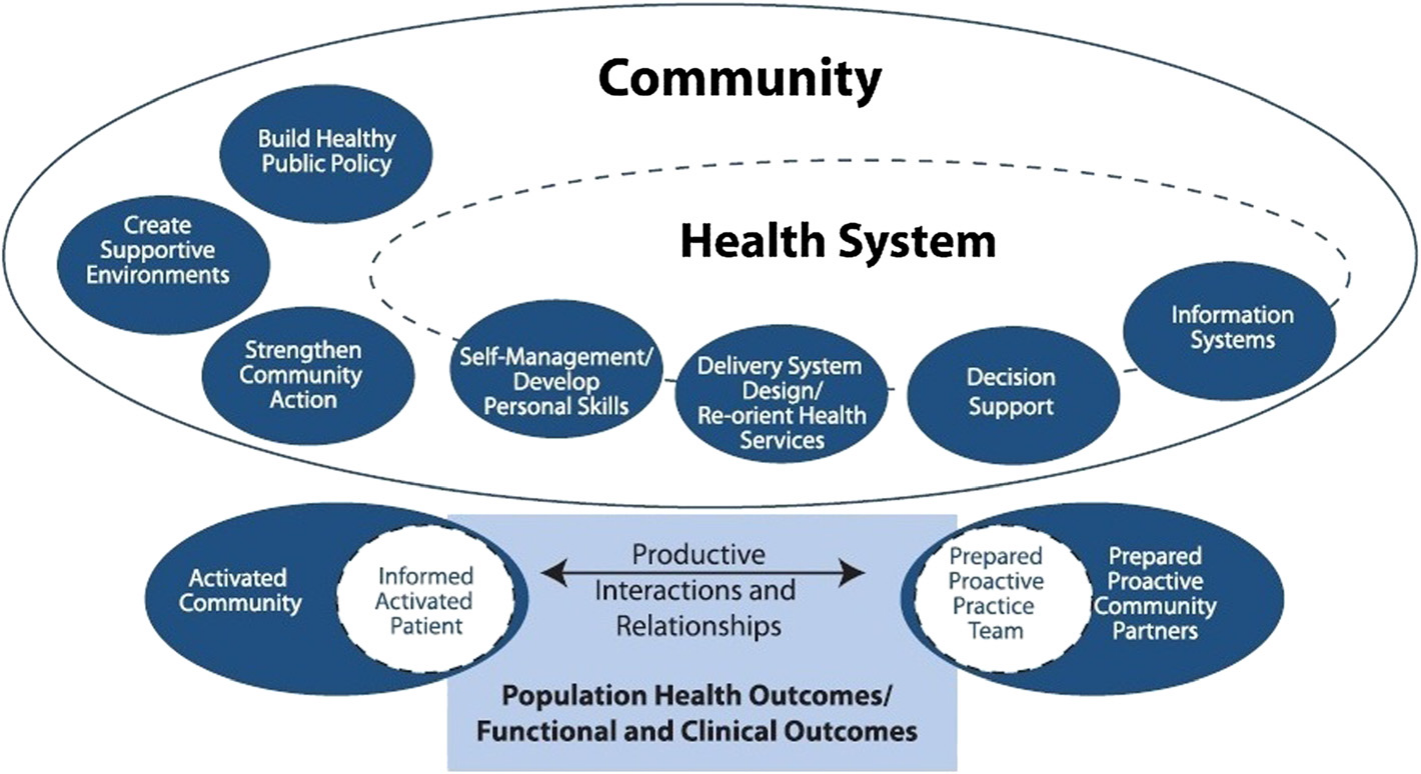
The Expanded Chronic Care Model. Obtained with permission from Barr VJ, Robinson S, Marin-Link B, Underhill L, Dotts A, Ravensdale D et al. The Expanded Chronic Care Model: an integration of concepts and strategies from population health promotion and the Chronic Care Model. Hosp Q. 2003;7(1):73–82

The ECCM [24] is a combination of the Chronic Care Model [32] and the principles of the Ottawa Charter [33] (see Fig. 1). Wagner et al. [32] proposed a re-design of the health system in response to the increasing number of patients with a chronic disease. Until then, the health system was focused on the treatment of communicable diseases. The Chronic Care Model shows how to provide appropriate care for patients with a chronic disease. It is characterized by the productive interactions and relationships between health professionals and patients [24, 32]. Patients have to become more responsible for their own health, and professionals have to adopt a proactive, supporting role to encourage patients’ health competencies [32, 34]. This is necessary because most of the unhealthy determinants influencing health reside outside the health sector [35]. Therefore, Barr et al. [24] added the principles of the Ottawa Charter to the Chronic Care Model in the ECCM. This created a focus on health promotion to construct supportive environments for citizens, thereby making them able to make better choices regarding their health in everyday life [33].

The ECCM complies with the multidisciplinary approach by combining population health promotion and the prevention and management of chronic disease [24]. The focus is broader than only persons with a disease; everyone and the whole community is addressed to live healthily [24, 36]. Health becomes central instead of illness [37] because of the interaction between the healthcare sector and other sectors in the community, such as transport, education, and sport. The ECCM visualizes the different stakeholders, actors and components involved in the connection between healthcare and health promotion. It gives insight into the broader health system wherein CSCs have to work and which components could be used to arrange the connection between primary care, sport and physical activity.

#### Theory of Triadic Influence

**Fig. 2:**
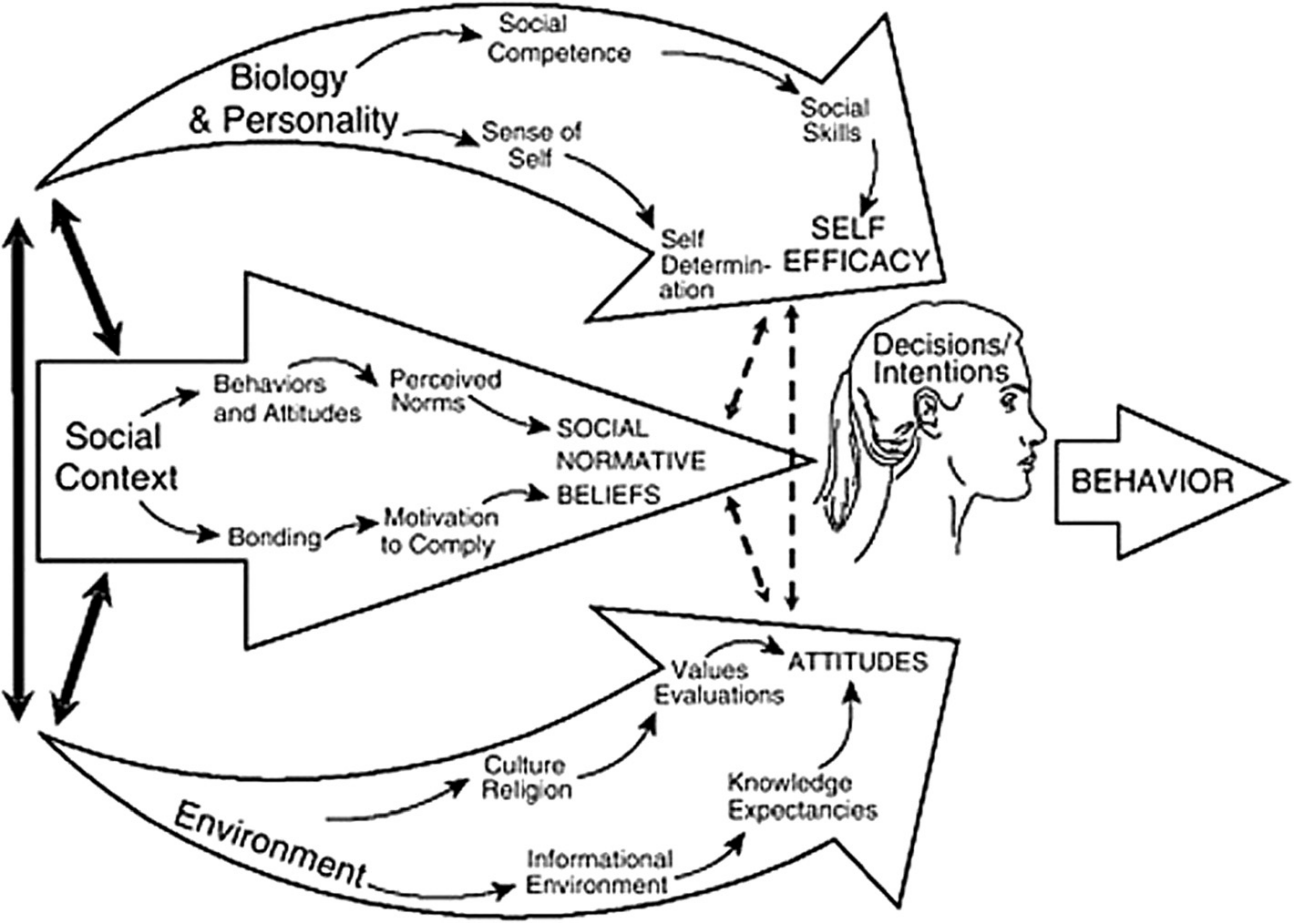
Theory of Triadic Influence. Obtained with permission from BR, Petraitis J. The Theory of Triadic Influences. A new theory of health behavior with implications for preventive interventions. Adv Med Sociol. 1994;4:19–44

TTI [31, 38] proposes that behaviours are most immediately controlled by decisions or intentions (see Fig. 2). These decisions and intentions to perform behaviours result from an individual’s attitude towards behaviour, social normative beliefs, and self-efficacy behavioural control [31]. It is a comprehensive theory, in which other theories with a focus on different aspects of the whole have been brought together [39]. Variables are organized along two dimensions: *levels of causation and streams of influence*, structured in a logical 3 × 3 framework [39].

The TTI arranges variables by different levels of causation, wherein individual control decreases with a higher level: 1) proximal or immediate causes have direct effects on behavior, 2) distal or predisposing causes are mediated through other variables, and 3) some causes are underlying or ultimate causes that are broad and relatively stable [31]. These levels act through the proposed streams of influence, resulting in intentions and behaviors: 1) intra-personal influences that contribute to a person’s self-efficacy, 2) interpersonal social influences that contribute to social normative beliefs, and 3) cultural-environmental influences that contribute to attitudes [31, 39]. To elucidate how CSCs directly or indirectly influence residents’ physical activity behavior and to assess the impacts of CSCs, the TTI will be used. CSCs’ indirect influence stems from the initiated connection with other relevant stakeholders to connect primary care, sport, and physical activity and the preconditions arranged by national, regional, and local organizations to facilitate the work of CSCs (Study I). This will lead to changes in work structures, policies, and physical activity facilities and is shown and explained by TTI through the more distal causes and cultural and environmental influences. CSCs’ direct influence takes place through direct contact with the target group to arrange and perform lifestyle interventions (Study II). In the TTI, contact with the CSC is a proximal cause that will lead to changes in intra-personal, interpersonal, cultural, and environmental influences.

## Methods/design

### Study design

**Fig. 3:**
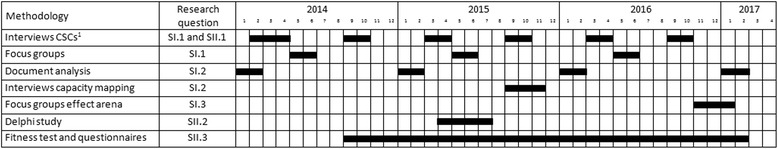
Overview of data collection methods and planning of the study. ^1^Care Sport Connectors

To study the role and the impact of the CSC, a longitudinal multiple case study will be conducted in nine municipalities spread over the Netherlands, from 2014 until the end of 2016. A mixed methodology will be used to perform action research and process evaluation. Fig. 3 provides an overview of the data collection methods and the planning of this study.

Action research provides direct feedback about the results to the CSCs and stakeholders, thus helping them to decide how to continue [40]. This is made possible by the use of tools which facilitate a learning process for CSCs and stakeholders in order to instigate change to improve practice [41]. Process evaluation will be used to monitor and document program implementation and can aid in elucidating the relation between specific program elements and program outcomes [42].

A mixed methodology is suitable for an appropriate multiple case study and action research design [43, 44]. Therefore, data will be collected through interviews, focus groups, document analysis, questionnaires, literature study, and a health-related fitness battery. These methods will be used in multiple rounds over three years to reveal changes over time. The fitness battery will be applied in a one group pre-test/post-test design, with a baseline measurement and two post-tests.

### Study population

Nine municipalities, spread over the Netherlands, were selected through convenience sampling based on project partners’ contacts. Inclusion criteria were: 1) the municipality has appointed a CSC for the next four years (until 2017) with the task of connecting primary care, sport, and physical activity and 2) the CSC’s target group is comprised of adults. The particular CSC was selected in consultation with the representative civil servant in each municipality; the total number of CSCs in the study is 14. This is approximately 15 % of the CSCs employed to connect primary care and sport for adults in the Netherlands [45].

Each research question has additional participants such as professionals in the alliances of CSCs, national policymakers, and experts in the field of health, sports, and physical activity policy, residents, primary care professionals, sports professionals, and participants in lifestyle interventions. These are explained in the description of each research question; an overview is shown in Table 1.

**Table 1:** Study overview – frameworks, methods, tools, participants, and repeats

Research question	Framework	Method	Tools	Participants	Repeats
SI.1		Literature review			1
	HALL framework	Interviews	Network Analysis Tool	14 CSCs^1^	6
			Timeline Method		
			Levels of Collaborations Survey		
		Focus groups	Coordinated Action Checklist	14 CSCs^1^ and professionals in their alliances	3
			Timeline Method		
SI.2	ADEPT model	Document analysis	Checklist based on ADEPT model		4
		Interviews	Local public health capacity mapping checklist	9 policymakers of the selected municipalities	1
SI.3		Focus groups	Effect arena	14 CSCs^1^, their alliances and residents	2
SII.1		Interviews		14 CSCs^1^	6
SII.2		Literature study			1
		Delphi study		Representative sample of the following professions; general practitioner, practitioner nurse, physiotherapist, coordinator social neighborhood team, public health policy coordinator, trainers, chairmen of sports clubs, CSCs^1^ and lifestyle programs participants	3–4
SII.3	Toronto Model	Fitness tests		Adults who participate in lifestyle programs arranged by, or with the help of, the CSC^1^	3
	Conceptual framework	Questionnaires			

^1^Care Sport Connectors

### Study I

Study I focuses on the expected alliances of CSCs and the preconditions which facilitate or hinder CSCs in forming these alliances. The study population will consist of intermediary target groups, such as CSCs, professionals in primary care, professionals in sport and physical activity who implement lifestyle programs, policymakers, and staff members of supporting organizations.

### SI.1: Connecting primary care, sport and physical activity, and the role of the CSC

To assess CSCs’ impact on participants’ environmental stream, CSCs’ role in forming alliances between primary care, sport, and physical activity will be studied, and factors that contribute to the collaboration in these alliances will be identified. Therefore, Koelen et al.’s [12] HALL framework will be used. The HALL framework identifies three clusters of factors that either hinder or facilitate the success of alliances: 1) institutional factors: the circumstances or incentives rooted in the institutional and economic environment of organizations that participate in the alliances, 2) personal factors of participants in the alliance, for example attitudes and beliefs, self-efficacy, social identity, and personal relationships, and 3) factors relating to the organization of the alliance, for example a flexible timeframe, roles and responsibilities, communication structure, management, shared mission, building on capacities, visibility [12].

### Methods and participants

#### Literature review

To our knowledge, there is no review available with a focus on intersectoral collaboration between the primary care and the sports sector in order to promote physical activity. Therefore, a review will be conducted with the aim of: 1) documenting and describing collaboration initiatives between the primary care and sports sectors in order to promote physical activity and 2) identifying barriers and facilitators in these collaboration initiatives between the primary care and the sports sector.

#### Interviews

To study the processes that contribute to the connection between primary care, sport, and physical activity and conditions that facilitate or hinder CSCs in their work, every six months, for three years, a semi-structured interview will be held with the 14 CSCs, in total 84 interviews. The topics will be based on the HALL framework and will relate to the level and functioning of the collaboration in the alliances, the role of CSCs, and preconditions for CSCs’ work.

#### Focus groups

To study the processes that contribute to the connection between primary care, sport, and physical activity, every year, for three years, a focus group will be held with the 14 CSCs and the professionals in their alliances. In total, 42 focus groups will be held. These focus groups will concentrate on the level and functioning of collaboration in the alliances.

#### Tools

In the interviews with the CSCs and the focus groups, we will use existing and validated tools that assess collaboration and at the same time facilitate discussion. These tools generate directly actionable knowledge. To study the processes that contribute to the connection between primary care, sport, and physical activity, Zaalmink et al.’s [46] Network Analysis Tool, Wagemakers et al.’s [47] Coordinated Action Checklist, Zaalmink et al.’s [46] Timeline Method, and Frey et al.’s [48] Levels of Collaboration Survey will be used.

#### Network analysis tool

The Network Analysis Tool [46] gives insight into a network’s involvement in a specific initiative. Roles of different contributors will be explored to become aware of their position in the network, thereby making it possible to get an overview of the network. This will lead to actionable knowledge because the CSC can decide whether the existing network has potential to grow and build further. This analysis reveals novel suggestions to bring the initiative one step further.

#### Coordinated Action Checklist

Coordinated action is the collaboration of two or more sectors to accomplish an outcome. The Coordinated Action Checklist [47] can be used for the facilitation and evaluation of community health partnerships with different contexts and levels, phase of the program, and participants. It evaluates collaboration for diverse dimensions, such as suitability of partners, task dimension, relation dimension, growth dimension, and visibility dimension. Results will be visualized to give insight into strengths and possible improvements, thereby encouraging feedback and discussion.

#### Timeline method

The Timeline Method [46] sorts out important events and influences inside and outside the network. It gives insight into these events and influences from each participant’s point of view in a certain time frame. This encourages discussion and evaluation to facilitate the collaboration in a positive manner.

#### Levels of collaboration survey

The Levels of Collaboration Survey [48] gives insight into different stages of collaboration with a description of the networking, cooperation, coordination, coalition, and collaboration stages. The stage of collaboration at a given time will be represented by a score on the scale. To measure changes over time, scores will be compared over time.

### SI.2: Conditions at national and local level that facilitate or hinder CSCs

CSCs work in different municipalities and therefore the contexts in which they work will differ. This will lead to differences in the way CSCs operate in forming alliances and to differences in the way local residents’ environmental stream and their physical activity behaviour will be influenced. For that reason, the conditions in national and local policy and the public health capacity of the municipalities will be assessed to identify the context in which each CSC works. Also, CSCs’ experiences with the preconditions for their work will be addressed during the interviews to identify facilitators and barriers.

### Methods and participants

#### Document analysis

To get insight into national and local policy regarding public health and CSCs, a document analysis will be performed every year. National policy and the local policy of each municipality will be analyzed with the use of a checklist based on Rütten et al.’s ADEPT model [49]. ADEPT, which stands for Analysis of Determinants of Policy Impact, aims to explain and influence policy development and policy impact implementation under four headings: goals, obligations, resources, and opportunities. The ADEPT model is useful to elucidate the role of policy processes in health promotion output and outcome. In addition, it is useful to identify necessary conditions in policy for a broker role such as the CSC.

#### Interviews

To assess the local-level conditions in which CSCs are working, policymakers from the nine municipalities will be interviewed. The local public health capacity mapping checklist, based on the frameworks of Meyer et al. [50] and Aluttis et al. [51, 52], the tools of Aluttis et al. [53] and Bagley and Lin [54], and interviews with experts, will be used during the interview to identify the context in which CSCs are working and the contextual changes over a period of time. This will make it possible to compare the capacity of the participating municipalities.

### Tools

#### Local public health capacity mapping checklist

This checklist consists of five dimensions to map the public health capacity of a municipality. These dimensions-policy characteristics, organizational structure, resources, programs and partnerships, and municipal context-are operationalized on the basis of the tools of Allutis et al. [53] and Bagley & Lin [54]. Quantitative operationalizations will be interrogated with a questionnaire prior to the interview wherein the qualitative operationalizations will be addressed. This will give the opportunity to clarify ambiguities in the questionnaire with the policymakers.

### SI.3: Mediated impacts and perceived societal benefits

Physical inactivity is an enormous risk factor for non-communicable diseases, which are currently creating an economic burden due to the increased prevalence of physical inactivity. Because the policy concerning CSCs—to connect primary care, sport, and physical activity and to prevent inactivity—is new and unique, it is necessary to evaluate the perceived benefits for the municipality, its residents, and professionals in the primary care and sports sectors.

### Methods and participants

#### Focus groups

To assess the impact of CSCs and the perceived societal benefits for the municipality, in total 14 focus groups with the CSCs, their alliances, and residents will be held at the end of the project. The aim of these groups is to identify the kind of programs conducted, the perceived results of these programs, and the perceived impact for professionals in the primary care and sports sectors, the neighbourhood, and its residents. The effect arena [55] will be used to structure these focus groups.

### Tools

#### Effect arena

The effect arena [55] structures the dialogue about an intervention’s investments and societal benefits as perceived by stakeholders. This is made possible by the completion of the following steps; problem analysis, determination of zero alternative, determination of project alternatives, identification of costs and effects, quantification and monetization of effects. It gives insight into and the possibility to examine the added value of an intervention; this is the first step towards a societal costs-benefits analysis.

### Data analysis Study I

The interviews and focus groups will be audio-taped and transcribed (intelligent verbatim style). The data analysis will be based on Creswell’s [56] six steps for qualitative data analysis. So, after the transcripts of the interviews and the focus groups are read, they will be coded and analysed using software for qualitative analysis (Atlas.ti, version 7.0). Both top-down and bottom-up coding will be used to analyse the interviews and focus groups. The top-down coding will use with predefined codes based on factors mentioned in the TTI and the HALL framework. The bottom-up coding (free coding) will trace general themes that emerge in the interviews and focus groups. In this way, relevant topics devised in advance of the study design and relevant topics from practice will be fully mapped. The codes will be clustered into themes. These themes will make it possible to interrelate and interpret the data [56].

The data gathered in the cases will be used to describe each case and build explanations on the CSC role in connecting primary care, sport, and physical activity [44]. In addition, we will make use of cross-case synthesis. This cross-case synthesis treats each individual case study as a separate study [44]. Word tables will be used to display the data from the individual cases according to the different frameworks presented in the theoretical framework. These word tables will also be used to analyse whether different groups of cases appear to share some similarities and deserve to be considered instances of the same type of general case [44]. Similar results in this study’s different cases will make it possible to draw general conclusions—for example, factors that hinder or facilitate the connection between primary care, sport, and physical activity.

### Study II

Study II will concentrate on lifestyle programs participants to identify health and physical activity behavior changes. In addition, the study will reveal facilitators for, and barriers to, the implementation of appropriate lifestyle programs.

### SII.1: Lifestyle programs and target groups

Lifestyle interventions aim to improve people’s health on themes such as smoking cessation, reduction of alcohol abuse, health dietary improvement, increased physical activity, or a combination of these themes. This study focuses on physical activity programs, and perhaps also programs in which other themes are also targeted.

Physical activity is defined as bodily movement produced by the contraction of skeletal muscle that increases energy expenditure above the basal level [57]. It can be classified in several ways, such as purpose, intensity, and type. The Dutch Healthy Physical Activity Guideline, which is 30 min of moderate physical activity at least five days a week, takes duration, frequency, and intensity into account [4]. Other elements that are crucial to stay physically active in the long term, revealed in previous studies, are cost, trainer qualifications, environment, and point in time [18, 19, 23, 58]. These elements are relevant to describe in a monitor report, and, additionally, relevant elements described in Wolfenstetter’s conceptual framework [59] will be monitored to create input for a societal costs-benefits analysis.

### Methods and participants

#### Interviews

Interviews will be conducted with the CSC to retrieve information about all the lifestyle programs arranged by, or with the help of, the CSC. During the interview, a table with characteristics of the lifestyle programs will be filled in to get insight into the type of programs and their target groups. These interviews will be held every six months with the 14 CSCs, in conjunction with the interviews of for research question SI.1.

### SII.2: Strategies and preconditions to increase participation, self-management and transferal of primary care patients

As mentioned before, principles such as participation, transferal of patients from primary care to local physical activity facilities, and enhancing self-management are essential but hard to realize in lifestyle programs. This part of the study will focus on these principles to improve the implementability of lifestyle programs.

### Methods and participants

#### Literature study

To gain input for a Delphi study, a literature study will be conducted to get insight into: 1) the different levels of participation and the experiences with community participation in lifestyle interventions, 2) indicators of self-management for individuals with, or at risk of, chronic disease, 3) experiences with the transferal of patients from primary care to local physical activity facilities in the Netherlands, and 4) views and experiences of individuals with, or at risk of, chronic disease on behavior change techniques used to enhance physical activity adherence during, and the maintenance after, a program. In addition, the review of research question SI.1 will be used to get insight into facilitators and barriers for intersectoral collaboration between the primary care and sports sectors.

#### Delphi study

A Delphi study will be carried out to identify strategies to increase participation, self-management, and transferal of patients. The ultimate aim is that this knowledge will contribute to the development of appropriate lifestyle interventions and their implementation. In order to get a comprehensive insight into the barriers and challenges to these different strategies, professionals representing different sectors will be involved in the Delphi study. From each of the following professions, a representative sample of Dutch professionals will be included by random sampling: general practitioner, practitioner nurse, physiotherapist, coordinator of the social neighborhood team, public health policy coordinator, trainers, chairman of a sports club, and CSCs. Additionally, participants of lifestyle programs will be included: 10 potential participants, 10 dropouts, and 10 participants who have completed a lifestyle program.

The first round of the Delphi study will consist of an open-ended questionnaire to discover viewpoints of each profession and participants in relation to physical activity, lifestyle programs, and intersectoral collaboration for the transferal of patients, self-management, and participation. This will reveal opportunities and barriers for the implementation of certain programs, and these will be the input for the statements in round 2. The statements in round 2 will be scored on a 7-point Likert scale by each professional and participant in order to determine which tasks will be recognized as their responsibility to ensure a better implementation of lifestyle programs and the intersectoral collaboration between the health and sports sectors for the transferal of patients. In the third and optional fourth round, there needs to be consensus about the possibilities within each profession and the necessities for each profession. The statements on which consensus is reached will be used to compose a checklist for CSCs to develop and implement lifestyle programs.

### SII.3: Effect in terms of physical activity behavior and maintenance, self-management, quality of life, experienced health, and health gains

The defined outcome of the CSC role is an increased number of residents participating in local sports facilities and being physically active in their own neighbourhood, for which it is necessary that people have the possibility to maintain their physical activities. This goal is proposed because of the increase in non-communicable diseases partly caused by inactivity. It is necessary to know whether the lifestyle interventions arranged by, or with the help of, CSCs influence physical activity behaviors and consequently improve people’s health. Therefore, this part of the study will take the effects of lifestyle interventions into account.

The complex relationship between physical activity, fitness, and health is described by Bouchard and Shephard in the Toronto Model [60]. A basic level of fitness is required for overall health in all individuals, the so-called health-related fitness, defined as ‘an ability to perform daily activities with vigor’ and a lower risk of developing non-communicable diseases. Health-related fitness is divided into five components: morphological, muscular strength and endurance, motor, cardio-respiratory fitness, and metabolic fitness. Physical activity influences health-related fitness directly and consequently has an impact on an individual’s health. The components of health-related fitness are measurable and will form the basis of the fitness test.

However, the concept of health is broader than the measurable components of health-related fitness. Huber et al. [61] introduced the dynamic definition of health as ‘Health as the ability to adapt and to self-manage, in the face of social, physical and emotional challenges’. In 2013, they operationalized the concept into the measurable dimensions of body functions, mental functions and perceptions, spiritual dimension, quality of life, social participation, and daily functioning. This conceptual framework will be used in the current study to decide which topics are relevant to include, in relation to physical activity and health, for a questionnaire to assess the impact on health.

### Methods and participants

In a one-group pretest/post design, lifestyle programs participants will perform a fitness test and fill in a questionnaire to assess their maintenance of physical activity and health gains. This will take place at the start of the lifestyle program (T0), at the end of the lifestyle program (T1), and one year after the start of the lifestyle program (T2).

#### Fitness test

The fitness test includes all components of health-related fitness by measuring blood pressure, heart rate, height, weight, percentage fat, waist circumference, flexibility of the hamstring, shoulders and back, arm and leg strength and endurance, dynamic balance, blood glucose, cholesterol, and cardio-respiratory endurance. To ensure that it is safe for them to participate in the fitness test, potential participants must first pass the Par-q questionnaire.

#### Questionnaire

The questionnaire will retrieve information about physical activity behavior, sedentary behavior, stages of changes, experienced health, self-management, motivation, self- efficacy, experienced health gains, knowledge, goals, and healthcare use.

#### Sample size calculation

The study population of the one-group pretest/ post-test will consists of adults who start a lifestyle program arranged by, or with the help of, the CSC in one of the nine municipalities. Inclusion will be determined by convenience sampling. The sample size calculation is based on alpha = 0.05 and power = 0.80 and assumes additionally:

- The primary outcome measure is maintenance of physical activity behavior after a lifestyle program, by participating in a local sports or exercise activity. Pilot data from the BeweegKuur revealed that 10–30 %, depending on the context, of the participants continue participating in local sports facilities after the lifestyle program [S.P.J Kremers, Personal Communication, November 2012]. The expected value for this study is 40 % because of the introduction of the CSC, although 20 % is set as the criterion for this sample size calculation.
- Some of the effects will be explainable by differences in support, lifestyle programs, and context in each neighborhood. There are no data available from the BeweegKuur about the intra-class correlation coefficient (ICC). An ICC of 0.15 is assumed, based on research into common values and similar interventions [62–65].

The required number of participants is 414, which will lead to 258 independent observations due to a design effect of 1.6. On the assumption of a drop-out rate of 10 % during the lifestyle program [66], 10 % at T1, and 20 % at T2 [67], a baseline sample of 640 participants is required.

### Data analysis Study II

The first round of the Delphi study will be analyzed via content analysis. Similar answers will be combined into themes with the use of Atlas.ti 7.0, and each theme will be used to generate statements. The statements, scored on a 7-point Likert scale, in the subsequent rounds will be analyzed with the help of the SPSS program. Measures of central tendency and level of dispersion will be used. Agreement is reached if 80 % of respondents’ responses fall within two categories on a 7-point Likert scale.

The quantitative data gathered via the fitness tests and questionnaires will be analyzed using the SPSS program. Multivariate techniques make it possible to test whether there is a relation between dependent variables such as physical activity behavior or motivation and independent variables such as gender, age, and nationality. Longitudinal data analysis will be used to study the individual development of the outcome variables and to determine whether there is a relationship with the individual development of other variables.

## Discussion

This study is designed to provide information about the implementation of the CSC function in nine municipalities, spread over the Netherlands. Through a mixed methods design, it will be possible to examine whether CSCs can achieve their objectives: 1) to connect primary care, sport and physical activity and 2) to increase the number of physically active residents. Besides this, the study will reveal whether a broker role contributes to improved intersectoral collaboration, increased participation by residents, transferal of patients from primary care to physical activity facilities, and self-management. Facilitators and barriers that are brought to light will be transformed into practical tools for CSCs and recommendations for supportive organizations to advance health promotion theory and practice in the Netherlands.

### Relevance

The number of people with one or more chronic diseases is increasing, and this trend could be reduced by promoting physical activity. A healthier population will benefit society at large through, for example, enhanced wellbeing, a decrease in healthcare costs, and higher work productivity [68]. The CSC function is implemented under national and local policy in order to reverse this trend, and this study will reveal whether CSCs are able to fulfill these policy aspiration.

The novelty of the CSC function means that there is no clear job description with required competencies. Therefore, it is not yet clear for what CSCs can and should be held accountable. By the use of qualitative and quantitative methods in multiple cases, it is possible to get insight into results achieved, opportunities, and realistic expectations. In addition, this study will generate actionable knowledge with stakeholders to improve practice immediately. Process evaluation makes it possible to get insight into developments achieved because of the implementation of this actionable knowledge. Accurate actionable knowledge is very helpful for a new, developing function, especially because of the expanding number of Neighborhood Sport Coaches in the Netherlands. At the start of this study in 2012, 3.2 % of the 1850 FTE for Neighborhood Sport Coaches were employed as CSCs for adults in the Netherlands [69]. An in-depth study over 18 municipalities revealed that, in 2014, 12 % of the surveyed Neighborhood Sport Coaches focused on connecting primary care and sport [70].

### Strengths and limitations

The study design is optimized for internal and external validity because action research, process evaluation, and the one-group pretest/post design are combined. The principle of triangulation is continuously applied to optimize the reliability of this study, using multiple methods, multiple sources, and multiple cases [71, 72]. Internal validity is enhanced by triangulation of methods and resources, whereby results will be checked with other stakeholders. In addition, other verification techniques will be used, such as expert consultation and loop learning [43, 73]. External validity is enhanced by studying multiple cases. Case studies take place in real-life settings and provide insight into the why and how in practice. Similar results in different cases will make it possible to draw general conclusions. This will result in formulated preconditions and prerequisites for CSCs rather than the general effect of CSCs. Because of differences in contexts in the multiple cases and the absence of a control case without a CSC, the latter is not possible.

In this study, a one-group pretest /post design will be used to measure physical activity maintenance and health gains. This design is appropriate to follow patients and residents who participate in activities and interventions, organized by, or with the help of, the 14 CSCs in this study, over an extended period. Individual-level results obtained from the one-group pretest/post design will be linked with results at the intervention, the environment and the policy level in order to be able to explain why changes in physical activity behavior and health have taken place, or not. For this study, a randomized controlled trial design is not appropriate, because it is impossible to arrange suitable control groups in real-life settings that resemble the contexts of the cases in our study. The context is different in each case and is exposed to continual change over time because of the action of the CSC, but probably also because of other stakeholders and events outside the control of the study [74]. Linking and integrating results gained by multiple methods at different levels will result in a validated assessment of the impact of CSCs on connecting the primary care and sports sectors, changes in residents’ physical activity behavior will be ascertained and the circumstances in which this will happen will be established [75]. The assessment, in combination with general lessons learned from the different case studies, will make it possible to determine whether CSCs are able to fulfill the policy aspiration and whether it would be beneficial to extend this function over more municipalities.

### Ethics approval

This study has been approved by the Medical Ethical Review Committee: CMO Regio Arnhem-Nijmegen (file number 2013–492).

## Abbreviations

ADEPT: Analysis of determinants of policy impact
CSC: Care Sport Connector
ECCM: Expanded Chronic Care Model
SI: Study I
SII: Study II
TTI: Theory of Criadic Influence
